# Depth discrimination of constant angular size stimuli in action space: role of accommodation and convergence cues

**DOI:** 10.3389/fnhum.2015.00511

**Published:** 2015-09-16

**Authors:** Abdeldjallil Naceri, Alessandro Moscatelli, Ryad Chellali

**Affiliations:** ^1^Department of Cognitive Neuroscience, Cognitive Interaction Technology Center of Excellence (CITEC), Bielefeld UniversityBielefeld, Germany; ^2^Nanjing Robotics Institute, College of Electrical Engineering and Control Science, Nanjing Tech UniversityNanjing, China

**Keywords:** depth discrimination, constant angular size, virtual environments, real environment, accommodation, convergence

## Abstract

In our daily life experience, the angular size of an object correlates with its distance from the observer, provided that the physical size of the object remains constant. In this work, we investigated depth perception in action space (i.e., beyond the arm reach), while keeping the angular size of the target object constant. This was achieved by increasing the physical size of the target object as its distance to the observer increased. To the best of our knowledge, this is the first time that a similar protocol has been tested in action space, for distances to the observer ranging from 1.4–2.4 m. We replicated the task in virtual and real environments and we found that the performance was significantly different between the two environments. In the real environment, all participants perceived the depth of the target object precisely. Whereas, in virtual reality (VR) the responses were significantly less precise, although, still above chance level in 16 of the 20 observers. The difference in the discriminability of the stimuli was likely due to different contributions of the convergence and the accommodation cues in the two environments. The values of Weber fractions estimated in our study were compared to those reported in previous studies in peripersonal and action space.

## Introduction

Various visual cues contribute to the perception of depth in humans (Bruno and Cutting, 1988; Nagata, 1991; Landy et al., 1995; Ware, 2004). According to several studies (Bruno and Cutting, 1988; Landy et al., 1995), the combined depth estimate is based on a weighted average of these multiple cues. If the weight of each cue is proportional to the precision of the signal, the combined estimate would be statistically optimal (Landy et al., 1995). The relative weight of the different cues (and therefore their contribution to the combined depth estimate) changes depending on the target distance from the observer (Cutting and Vishton, 1995). For example, the visual depth sensitivity for accommodation, convergence, and binocular disparity drops at distances beyond 5 m (Nagata, 1991). Based on the weight of different cues, it is possible to divide the perceptual space into a peripersonal space (within arm reach or slightly beyond), an action space (from approximately 1 m to 30 m) and a vista or far space (beyond 30 m; Cutting and Vishton, 1995; Previc, 1998; Armbrüster et al., 2008; Naceri et al., 2011). The significance of the action space is that it is beyond arms reach and requires whole-body motion for interaction with it.

The angular size, which is the size of the image that an object produces on the retina of the observer, conveys important information about visual depth (Palmer, 1999). The angular size of the object, *a*, is related to the distance from the observer, *d*, from the well-established equation:

(1) $$\tan\;(a)=\frac{h}{d}$$

where *h* is the physical size of the object. The angular size of the object can only convey information about the distance to the observer provided that the physical size, *h*, is known. In order to use the angular size as a relative depth cue, observers use a heuristic that two otherwise identical objects in the visual scene have the same physical size. Therefore the relative distances can be determined from the relative size of the two objects (relative size cue), so that the object projecting the smaller retinal image is perceived as the one farther in space. A daily-life example of the relative size cue is the depth information provided by identical columns in a colonnade. Similarly, in our daily life experience, most objects do not inflate or shrink and the relative size of the same object at time t_0_ and t_1_ provides a relative distance cue (size change cue). For instance, a moving object whose retinal size increases over time is perceived as approaching the observer. Finally, for many familiar objects, an adult man, a car, a tree or a door for example, the physical size is roughly known and the equation can be solved with a certain approximation (familiar size cue).

The three angular size cues (familiar size, relative size and size change) are precise depth cues; accordingly, the presence of one of the angular size cues dramatically decreases the relevance of other sources of depth information (Tresilian et al., 1999; Kaufman et al., 2006). Several studies evaluated depth perception controlling for the relative and the familiar size cue, in both peripersonal (Rolland et al., 1995; Mon-Williams and Tresilian, 2000; Viguier et al., 2001) and action space (Allison et al., 2009). Rolland et al. (1995) investigated depth perception for a range of stimuli of 0.8–1.2 m. Observers compared the depth of two objects having different shapes (a cylinder and a cube); thereby the relative size did not provide a cue to the task. The authors evaluated the task in both real and virtual environment. The discrimination threshold, which is an inverse function of the precision of the response, was equal to 2 mm in the real environment and 15 mm in the virtual environment. According to the authors, “although the subject could not compare the relative sizes of the two objects to compare depth, they might still have used the size change in trying to assess depth”. A task similar to Rolland et al. (1995) was tested by Allison et al. (2009) for a standard stimulus placed at 9 m distance from the observer. The authors used two different objects for the standard and the comparison stimulus (a textured surface and a rod), therefore controlling for the relative size cue but not for the size change of the comparison. Other studies (Mon-Williams and Tresilian, 2000; Viguier et al., 2001) controlled for the size change by maintaining the angular size of the comparison object constant across trials. To the best of our knowledge, this paradigm has never been investigated in action space, for distances beyond 1.2 m from the observer.

Here we characterized depth discrimination in action space (1.4–2.4 m) in a forced-choice task where the angular size cue was accurately controlled. This was achieved by increasing the physical size of the target object as its distance to the observer increased. The aim of the study was to measure the discriminability of the stimulus when the size change cue was not available to the observer. In order to estimate the weight of the size change cue, we compared our results with previous studies in the literature, in both peripersonal and action space.

A second aim of the study was to estimate the role of the congruence between accommodation and convergence cue in action space, when the angular size was accurately controlled. To this end, we replicated our paradigm in both the real world and in a virtual reality (VR) setting. In VR, binocular disparity (stereopsis) and convergence were the only effective depth cues available to the observers. Noticeably, in all VR setups the accommodation and the convergence cues are in conflict, since observers accommodate at the screen level. This phenomenon is known as the convergence-accommodation conflict (Mon-Williams and Tresilian, 2000; Banks et al., 2013). Instead, the two cues were not in conflict in the real-environment task. Accordingly, previous studies showed that, in peripersonal space, the discriminability of depth is significantly worse in VR compared to the real environment (Rolland et al., 1995). However, the convergence-accommodation conflict has never been fully evaluated in action space. Previous studies reported that sensitivity to accommodation and convergence drops at a distance of beyond 5 m from the observer (Nagata, 1991). Therefore, it is important to evaluate these two cues beyond peripersonal space (Rolland et al., 1995) as their contribution to depth discrimination might change as the distance to the observer increases. To this end, we compared the discriminability of our stimuli between the two environments. If accommodation and convergence cues would provide the observer with a reliable cue to depth, we expect that the discriminability of the stimuli will be worse in VR (where the two cues are in conflict) compared to the real environment.

## Materials and Methods

### Equipment for Virtual Reality Setup

Two video projectors (model evo22sx+ from Projection Design) were placed side-by side on the ceiling of the experimental room and were equipped with two orthogonal circular polarization filters. Both beams were oriented towards a wide-screen and the projection distortion was corrected until two rectangular images (dimensions 1.805 × 1.535 m^2^) were perfectly overlapping. The observers’ head position was tracked using the WorldViz® PPT™ optical motion tracking system with six cameras (H series, update rate 175 Hz, latency < 20 ms). The head tracking allowed us to extract the actual position of subjects’ eyes, which was used for rendering the virtual scene.

**Figure 1:**
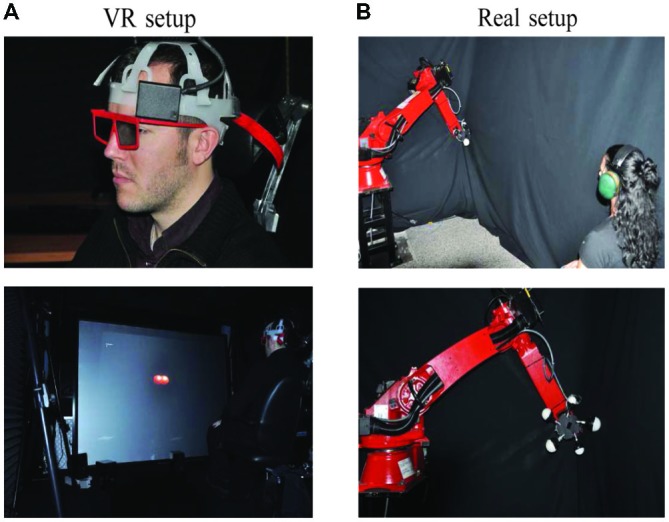
**Experimental setups for both real environment and virtual reality (VR) settings.** **(A)** VR setup. **(B)** Real environment setup. The robot arm has been covered with black material during the experiment and was not visible to the participant.

The left and right images of resolution 1280 × 1024 were generated using the library OpenGL® from a NVIDIA™ Quadro™ FX 3800 graphics card on a PC Dell® (Intel® Quad Core(Q7600), 2.4-GHz, 2-GB RAM) running under Linux operating system and were displayed simultaneously at 50 Hz. The observers, wearing light passive polarized glasses, were seated 2.2 m in front of the projection screen (see Figure 1A) so that visual accommodation occurred at approximately the same screen distance. A dark gray background was used in order to minimize undesirable objects’ ghost in the projected scene. Observers were asked to maintain their head position fixed on the chair headrest (Figure 1A) and to face towards the screen, which resulted in both eyes being positioned in the coronal and axial planes.

### Equipment for Real Object Setup

We designed a mechanical device (referred as “the juggler” later in the article) that was attached to a robotic arm: Comau® SMART™ SiX (6 axes, 6 kg payload, 160 kg robot mass; Figure 1B). The juggler allowed differently sized spheres to be presented at specific distances to the observer, allowing us to experimentally control the angular size of the real object. The device could display (or hide) up to five real spheres using a stepper motor. A black material was used to cover the robot parts and the spheres were illuminated with Light Emitting Diodes (LEDs) in an otherwise dark room (therefore the juggler and the robot arm were not visible by the observer).

Specifically, LEDs were placed inside the spheres, which were assembled using two parts. The front half sphere (the viewed part) was transparent and the back half sphere part was covered from inside with aluminum paper and black tape. This was done to minimize the use of any cues other than the ones we were interested. As for the VR experiment, observers were asked to maintain their head position fixed on the chair headrest (not shown in the figure).

### Task and Stimulus

**Figure 2:**
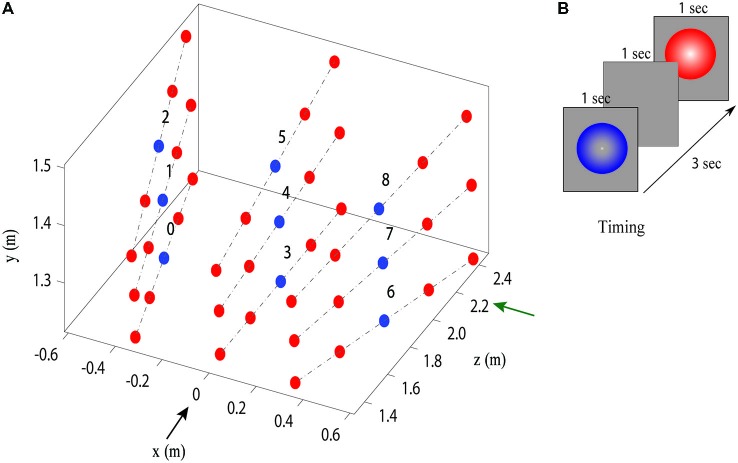
**Stimulus locations and timing. (A)** Stimulus lines enumerated from 0 to 8. The black arrow represents the direction of the observer’s view and the green arrow represents the big screen position for the VR setup. Red points represents comparisons and blue points represents standards. **(B)** Timing during one trial of the standard and comparison located at the same stimulus line.

Before each experiment, the participant’s eye height, eyes distance from the screen and interpupillary distance (IPD) were measured using a digital precision optical instrument. These measurements were used to set the parameters for the VR stimulus presentation. Lighting and shading were held constant for the stimuli presented in VR. In both VR and real objects experiments, participants were presented with a sequence of two spheres, one blue (standard) and one red (comparison) located along the same line in depth (Figure 2A), and then asked to verbally indicate which appeared closer by saying “red” or “blue”. Only one sphere was visible during each presentation interval, with one second pause between the two presentations (Figure 2B). The order of presentation (blue then red, or red then blue) was randomized and repeated 10 times. Each experiment consisted of two 20 min sessions, with a break between sessions. We manipulated both the distance and the angle (azimuth and elevation) at which the spheres were presented in the stimulus pair standard-comparison (Figure 2A illustrates the different angles tested in the experiment).

The blue spheres (the standard stimuli) had a diameter of 7 cm and were presented at a distance of 1.9 m from the observer, in one of nine possible positions in space (see Figure 2A). The red spheres were used as the comparison stimuli and were displayed at 45 possible positions with radial distance ranging from approximately 1.4–2.4 m with a step of 25 cm from the observer. The physical size of the red comparison spheres co-varied with distance so that participants observed a constant angular size, equivalent to the blue reference spheres (2.11°), in all positions.

### Participants

Twenty observers participated in the virtual environment study (11 males and 9 females; average IPD 6.22 ± 0.32 cm) and twelve new observers participated in the real environment study (six males and six females; IPD 6.20 ± 0.29 cm). All had normal or corrected to normal visual acuity. All participants in the VR experiment passed the Titmus stereo test. The Ethics Committee of the Italian Institute of Technology approved both real and virtual environments experiments. Informed written consent was obtained from all participants involved in the study. The participants that appear in Figure 1 have seen this manuscript and figure and have provided written informed consent for publication.

## Data Analysis

We applied Generalized Linear Mixed Models (GLMMs) to fit the data (Agresti, 2002; Moscatelli et al., 2012). GLMM is an extension of the ordinary General Linear Model, which allows the analysis of clustered categorical data (here, each cluster corresponds to the repeated measures from a single participant). In each trial, we considered the response variable *Y* = 1 if the observer reported that the comparison (red) was at a farther distance than the standard (blue), and *Y* = 0 otherwise. The predictor variable X in Model 1 is the distance in space of the comparison stimulus (indicated by the disparity-driven convergence cue in VR). By applying the Probit link function (i.e., the inverse of the cumulative standard normal distribution), Model 1 is the following:

(2) $${\text{Φ}}^{-1}[P(Y_{ij}=1)]=\alpha +\beta X_{ij}+Zu_{i}+\varepsilon_{ij}$$

Where *Y_ij_* and *X_ij_* are the response and predictor variables for observer *i* and trial *j*, and *α* and *β* are the fixed-effects parameters, common to all observers. The model assumes two different error terms; *ε_ij_* which accounts for the within-subjects variability and *u_i_* which accounts for the between observers variability. The product Z*u_i_* is usually referred as the random predictor.

The rationale for using a GLMM rather than Analysis of variance (ANOVA) is that the latter assumes a normally distributed response variable, which instead was binary (zero or one) in our case. In the model reported in the equation above, the fixed-effect parameter *β* (the slope of the linear predictor) estimates the precision of the response: the higher the parameter *β*, the higher the precision. The Just Noticeable Difference (JND = 0.675/*β*) is a measure of noise, the higher the JND the higher the noise in the response. The Point of Subjective Equality (PSE) estimates the accuracy of the response. We computed the PSE as PSE = −*α/β*. The PSE is the value of the test stimulus corresponding to a probability of response at chance level 0.5. In our experimental procedure, the response is accurate if the PSE is not significantly different from zero. We do not expect any bias in our experimental protocol, as the change in physical depth was the only relevant difference between the comparison and the reference stimulus. Testing the PSE is only an internal control here. We estimated the confidence interval of the PSE and the JND as explained in Moscatelli et al. (2012).

In each experiment, we evaluated three possible *nested models*: each model was applied separately in the real and virtual environments. In Model 1 and 2 we used only the change in depth as fixed-effect predictor variable. Model 1 (VR: modelVR1, Real environment: modelR1) includes two fixed effect parameters accounting for the intercept and the slope of the linear predictor and a single random intercept of equation 2. Model 2 (VR: modelVR2, Real environment: modelR2), includes the same parameters of model 1 and also a second random predictor (random slope). Model 3 (VR: modelVR3, Real environment: modelR3) includes the two random effects parameters and three-fixed effect predictors: the changes in depth, the changes in the azimuth and the elevation angles of the stimulus locations. The rationale of Model 3 was to test the impact of the azimuth and elevation line on observer performance. We compared the models by means of the Akaike information criterion (AIC; the best model is the model with the smallest AIC) and Likelihood Ratio test (LR test).

## Results

### Virtual Reality

**Figure 3:**
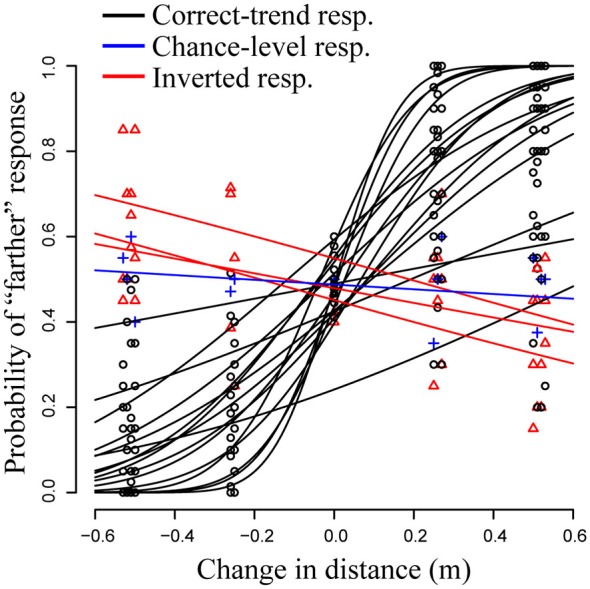
**Generalized linear mixed model (GLMM) fit and count data (*N* = 20) in the VR experiment.** One participant responded at chance-level, irrespectively of the change in the stimulus (in blue in the plot). Three participants produced a paradox response, i.e., they reported as farther in space the closer stimuli and* vice versa* (in red in the plot).

The AIC was smaller in modelVR2 (AIC2 = 1335.1) than in modelVR1 (AIC1 = 3003.9) revealing large differences between the observers in the discriminability of the stimuli (Figure 3). The LR test confirmed that modelVR2 provided a better fit to the data than modelVR1 and modelVR3 (*p* < 0.001). On the other hand, the values of the AIC were similar between modelVR2 and modelVR3 (AIC2 = 1335.1, AIC3 = 1335.7) and the difference between the two models was not significant at the LR test (*p* = 0.07). The non-significant improvement in model fit between modelVR3 and modelVR2 indicates that the azimuth and elevation angles (stimulus lines) do not affect observers’ performance. Based on these results, we selected the model modelVR2 for further analysis.

Figure 3 shows the observers’ proportions (circles) and the fitted GLMM (modelVR2; solid black lines). The fixed effect accounting for the slope was highly significant (*β* = 2.63, *p* < 0.001). This means that on average the observers were able to discriminate a change in the depth of the stimuli. The JND was equal to 0.26 ± 0.05 m (JND ± Standard Error; 95% Confidence Interval: 0.15–0.36 m), corresponding to a Weber fraction of 0.13 (the Weber fraction being the ratio of the JND and the standard stimulus). As mentioned above, the model revealed a high variability in the slope parameters between the observers (variance of the random slope = 6.22). The predicted slope was greater than 0 in 16 of the 20 observers. In three observers the *β* parameter was significantly smaller than zero (red triangles and lines in Figure 3); the significance of this finding was further evaluated by the single-subject analysis (psychometric functions), which confirmed the significance of the negative slope (*p* < 0.05). This means that these three participants produced a paradoxical response and perceived as closer in space the stimuli that were physically further in space, and* vice versa* (data in red Figure 3). In one observer (data in blue Figure 3) the parameter *β* was non-significantly different from zero (psychometric function: *p* > 0.05), that is, the response was at chance level irrespective of the depth of the comparison stimulus.

As expected, the PSE value was close to zero, thus, the responses were on average quite accurate (PSE = 0.03 ± 0.02 m; estimate and standard error).

### Real Objects Results

Similarly to VR data results, we compared three different models using AIC. The criterion was smaller in modelR2 (AIC2 = 639.31) than in modelR1 (AIC1 = 720.63). The LR test confirmed the better fit provided by modelR2 than modelVR1 and modelVR3 (*p* < 0.001). Values of AIC were almost the same between modelR2 and modelR3 (AIC2 = 639.31, AIC3 = 642.44) in accordance with non-significant LR test (*p* = 0.65). Therefore, we used modelR2 for further analysis.

**Figure 4:**
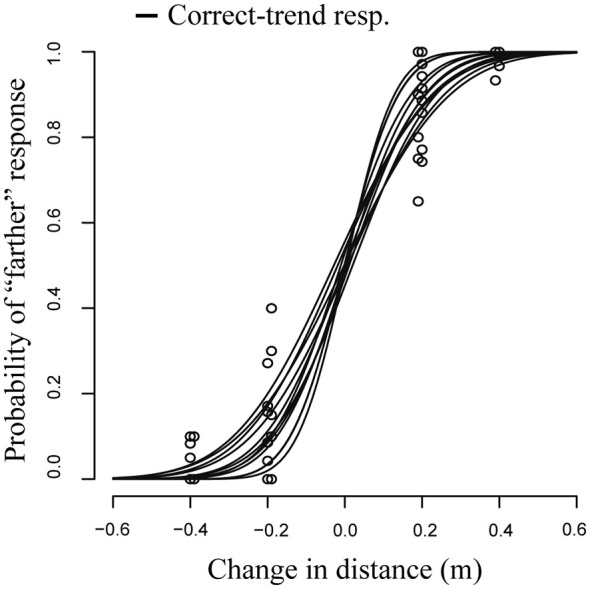
**GLMM fit and count data (*N* = 12) in real objects experiment.** The response was accurate and precise in all participants.

Figure 4 shows the model fit (solid lines) and the observed proportions (circles). The fixed effect accounting for the slope was highly significant (*β* = 6.82, *p* < 0.001), indicating a very high precision of the response. The JND was equal to 0.10 ± 0.01 m (JND ± Standard Error; 95% Confidence Interval: 0.08–0.11 m), corresponding to a Weber fraction of 0.05. In this condition, values of *β* were all significantly greater than 0 indicating that all observers were able to perceive depth, which indicates that participants did not rely on the target apparent size in their depth judgments.

As expected, the PSE was not significantly different from 0; thus, the responses were accurate (PSE = −0.0007 ± 0.005 m; estimate and standard error).

**Figure 5:**
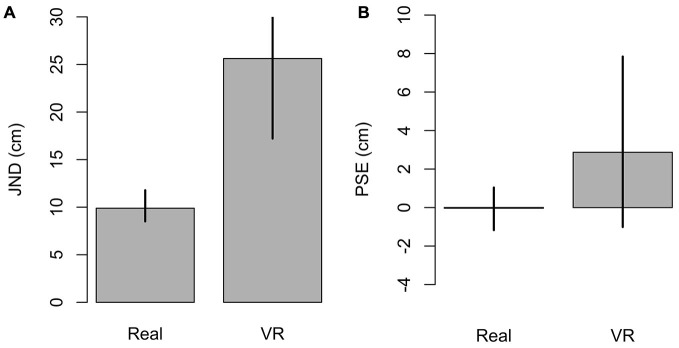
**Just noticeable differences (JNDs) and Point of subjective equalities (PSEs) in both real and VR setups, GLMM estimates. (A)** JND; the higher JND means a noisier response. **(B)** PSE, internal control, the response is expected to be accurate by the experimental design. Error bars are 95% confidence interval.

In the real environment accommodation and convergence provided a cue to depth, whereas in the virtual environment the two cues were in conflict since observers accommodated at the screen level. We compared the JNDs in real and virtual environments in order to evaluate the contribution of accommodation and convergence cues in action space. The predicted JND was about twice as large in VR condition compared to the real environment (Figure 5A). Crucially, 95% confidence intervals of the two JNDs were not overlapping, which means that the difference was statistically significant (*p* < 0.05). This finding suggests that the accommodation and convergence cues convey important information about depth in the tested stimulus range. Rolland et al. (1995) found similar results in peripersonal space. Our results extended their findings into action space. As expected for the PSE, we did not record any bias in both environments (Figure 5B), as the change in physical depth was the only relevant difference between the comparison and the reference stimulus.

## Discussion

**Figure 6:**
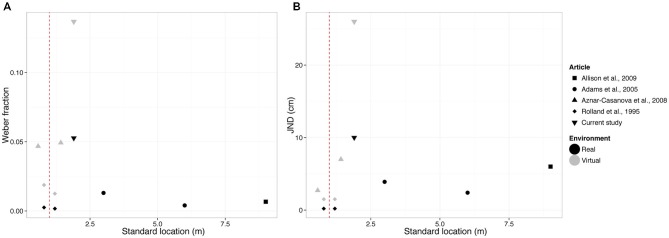
**Summary of mean weber fractions (panel A) and mean JNDs (panel B) of our study and other previous studies that did not control for the size change cue.** Red dashed line represents the end of peripersonal space and beginning of action space.

The perception of depth depends on the combination of multiple cues, whose reliability changes with the distance of the object to the observers (Gilinsky, 1951; Da Silva, 1985; Nagata, 1991; Landy et al., 1995; Loomis et al., 1996; Loomis and Knapp, 2003; Swan et al., 2007; Armbrüster et al., 2008; Saracini et al., 2009; Naceri et al., 2011). A cue of primary importance is the angular size, defined as the size of the image that an object produces on the retina of the observer. In our study, participants reported on their perceived depth of virtual and real stimuli located in action space, whose angular size was held constant between stimuli. The estimated JND was equal to 0.10 m in the real environment and 0.26 m in the VR setting, corresponding to a Weber fraction of 0.05 and 0.13, respectively. In Figure 6, we compared these values to previous studies in real and virtual environments where the relative change in the angular size across stimuli was not controlled and thus provided a cue to depth (size change cue). The discriminability of our stimuli was markedly worse, showing the important role of the size change cue in the tested range. This is further discussed in the following paragraph.

### Comparison with the Results of Previous Studies

As we explained in the introduction, it is important to distinguish between the relative size, the familiar size and the size change cues. These three cues are all related to the angular size cue, the only difference being the estimate of the physical size of the target object. Previous studies (Rolland et al., 1995; Allison et al., 2009) measured the discriminability of the stimulus in tasks where the relative or familiar size were not available. Yet the size change cue was always available and providing information on the relative depth. In order to estimate its weight in the discrimination of depth, we compared the weber fractions and JNDs of the current study with four other studies in peripersonal space (Rolland et al., 1995; Aznar-Casanova et al., 2008) and action space (Adams et al., 2005; Allison et al., 2009) where the size change cue was available. Values are summarized in Figure 6.

Specifically, Rolland et al. (1995) used standards at 0.8 and 1.2 m in real and virtual environments, Aznar-Casanova et al. (2008) used standards at 0.58 and 1.42 m in virtual environments, Adams et al. (2005) used standards at 3 and 6 m and Allison et al. (2009) at 9 m in real environment. The three angular size cues (relative, familiar and size change) were all available in the stimulus used in Adams et al. (2005) and Aznar-Casanova et al. (2008). Instead, Rolland et al. (1995) and Allison et al. (2009) asked participants to compare the relative depth of different, unfamiliar objects so that the familiar and relative size cues were not available. However, as discussed by Rolland et al. (1995), the observer may have used the size change cue in their depth judgments. This was not the case in our study where participants could not use the size change cue in their distance judgments. Importantly, we found higher JNDs and weber fractions values in our study compared to previous studies (Figure 6). This emphasizes the importance of controlling for all the three angular size cues in the task.

The Weber fraction estimated in our study in VR was comparable with the ones reported in a previous study where the angular size was held constant (Svarverud et al., 2010; this study was not included in Figure 6, since authors controlled for size change cue). Svarverud et al. (2010) investigated object depth judgment in impoverished virtual environments. The measured Weber fractions are equal to 0.12 at 1 m, and 0.35 at 3 m distances, which is comparable with our current estimate (0.18). Instead, their estimated Weber fraction increases up to 0.65 at 5 m viewing distance. In our review of the literature, we found only two studies controlling for the size change cue in real-world environments (Mon-Williams and Tresilian, 2000; Viguier et al., 2001). In both cases the task was performed in peripersonal space. Participants provided the response with a matching task; therefore we were not able to compare the reported error with results of other studies (expressed in terms of Weber fraction and JND).

### The Discriminability of Depth in Real and Virtual Environment

Previous studies showed that convergence is an absolute distance cue, whereas accommodation provides ordinal depth information in peripersonal space (Mon-Williams and Tresilian, 2000; Mon-Williams et al., 2001; Viguier et al., 2001). Here, we compared the results in real and virtual environment so as to quantify the contribution of convergence and accommodation cues in action space. In the present study, all participants were able to discriminate the depth of the target in the real environment (the slope parameter in modelR2 was highly significant, *p* < 0.001) using the available depth cues, namely the convergence, binocular disparity and accommodation cues. The azimuth and elevation angles did not significantly affect the response. Our results are consistent with Aznar-Casanova et al. (2008) where they found that changes in azimuth angles did not affect depth perception.

The JND was about twice as large in the VR setting than in the real environment (Figure 5A), that is, the response was noisier in the former. The relatively noisy result in the VR setting is in accordance with the results of Naceri et al. (2011), which applied a similar paradigm, using an head-mounted display, in peripersonal space (within arm reach). In the VR setting the accommodation and the convergence cues were in conflict, since observers accommodated at the screen level. This phenomenon is known as the convergence-accommodation conflict (Mon-Williams and Tresilian, 2000; Banks et al., 2013). In contrast to this, the two cues were not in conflict in the real environment. This suggests that accommodation and convergence cues provide a reliable cue to depth information in action space.

In the VR setting, we also found larger between-participants variability in the response compared to the real environment (Figures 3, 4). One observer did not perceive changes in virtual object depth (response at chance level) and three observers perceived depth with inversion—i.e., they perceived as closer those stimuli father in space, and* vice versa*. Paradox responses, where some of the participants perceived depth with inversion, were also found in peripersonal space (Naceri et al., 2011). Instead, no paradox response was observed when the angular size of target varied across stimuli distance (Naceri et al., 2011).

Comparing depth perception in real and virtual environments is a challenging task, due to the limitations of immersive VR displays, including convergence-accommodation conflicts (Hoffman et al., 2008), limited field-of-view (Knapp and Loomis, 2004), lack of graphical based-realism (Thompson et al., 2004) and mismatches between the viewed world and the experimental site (Interrante et al., 2006). In our study, we ensured that the VR and real object stimuli were matched accurately. However, in addition to the difference in the accommodation cue that we already mentioned in the introduction, small differences in lightning between real and virtual stimuli (we used LED lightening in the real setup) can partially account for the difference in the response. Still, we attempted to minimize these differences by preventing any back-illumination of the target spheres in the real environment and ensuring that the robot arm was not visible to the observers.

The range of distances tested in the present study are particularly relevant for the design of visual displays because they represent the average optimal view distance between the observer and a 3D large screen (40–70 inches). In the tested range of stimuli (1.4–2.4 m), depth discrimination of constant angular size stimuli was significantly noisier in the virtual environment compared to the real environment. The difference was likely due to the convergence-accommodation conflict in the virtual environment. Previous studies reported that the contribution of the accommodation and convergence cues to depth judgments drops in action space (Nagata, 1991). Our results demonstrated that they provide important depth information for a stimulus distance ranging from 1.4 to 2.4 m. The accommodation-convergence conflict, which is a major limitation for the design of 3D virtual environments, accounted for the drop in the performance in virtual stimuli compared to the real world.

## Conflict of Interest Statement

The authors declare that the research was conducted in the absence of any commercial or financial relationships that could be construed as a potential conflict of interest.
